# The Prevalence of Hepatitis B Virus Infection in Mashhad, Iran: A Population-Based Study

**DOI:** 10.5812/ircmj.8200

**Published:** 2013-03-05

**Authors:** Mohammad Taghi Shakeri, Bahareh Foghanian, Hosein Nomani, Majid Ghayour-Mobarhan, Maryam Sadat Nabavinia, Sina Rostami, Mitra Ahadi, Zahra Meshkat

**Affiliations:** 1 Department of Biostatistics, Public Health School, Mashhad University of Medical Sciences, Mashhad, IR Iran; 2 Microbiology and Virology Research Center, Mashhad University of Medical Sciences, Mashhad, IR Iran; 3 Biochemistry of Nutritional Research Center, Faculty of Medicine, Mashhad University of Medical Sciences, Mashhad, IR Iran; 4 Department of Biotechnology, School of Pharmacy, Mashhad University of Medical Sciences, Mashhad, IR Iran; 5 Department of Biology, Faculty of Sciences, Ferdowsi University of Mashhad, Mashhad, IR Iran; 6 Department of Internal Medicine, Ghaem Hospital, Mashhad University of Medical Sciences, Mashhad, IR Iran; 7 Women's Health Research Center, Mashhad University of Medical Sciences, Mashhad, IR Iran

**Keywords:** Hepatitis B virus, Prevalence

## Abstract

**Background:**

Hepatitis B virus (HBV) infection is the most common and serious liver infection in the world. An estimated 350 million people are chronic carriers of this virus, of whom, more than 620,000 die from liver-related diseases annually. Due to the vaccination program, prevalence of HBV, particularly among the younger generation, is reported to have declined in recent years in Iran.

**Objectives:**

The aim of this study was to evaluate the prevalence of HBV infection in Mashhad, North-East of Iran.

**Patients and Methods:**

Three thousand one hundred and ninety eight (3198) individuals living in Mashhad were studied using cluster sampling method. HBV infection was determined by HBsAg ELISA commercial kit. Positive results were subjected for PCR using HBV-specific primers. HBeAg, HBeAb, and HBcAb-IgM ELISA tests were performed for HBsAg-positive samples.

**Results:**

Patients’ age ranged from 15 to 65 years (Mean = 35.54 ± 14.85). Thirty four (1.0%) of the subjects were positive for HBsAg, of whom, 2.9 % (1 of 34 cases) were also positive in PCR-based screening. ELISA tests for HBeAg, HBeAb, and HBcAb IgM were positive in one (2.9 %), 27 (79.4%) and one (2.9 %) cases, respectively.

**Conclusions:**

According to our results, HBsAg was positive in 0.53 of the total population. The prevalence of HBV infection was seemingly low in Mashhad; however, an upward trend was observed in older subjects probably due to successful HBV vaccination coverage in the younger generation. Continuous surveillance and periodic population-based studies are essential to monitor the prevalence of HBV infection in Mashhad in the future.

## 1. Background

Hepatitis B virus (HBV) is classified in Hepadnaviridae family and has a circular, partially double-stranded DNA (1).Virus replication occurs in the liver; however, specific proteins and antibodies of the virus are present in the blood of infected individuals. Some blood tests have been developed to detect these proteins and antibodies (2). Despite the availability of an effective vaccine against hepatitis B virus, its infection remains a major public health problem throughout the world. Approximately, 350 million HBV carriers are chronically infected (3). Chronic carriers of HBV have an elevated risk of developing cirrhosis and hepatocellular carcinoma (HCC) which leads to death of an estimated 0.5 to 1.2 million subjects annually (3-5) .Worldwide, HBV infection is considered to be the 10th leading cause of death (4). The principal way of transmission is through blood and blood products. Hemodialysis, shared needles among drug abusers, dental surgery, receiving blood or blood products, cupping, tattooing, ear and nose piercing practices and sexual exposure to HBV can elevate the risk of transmission (6). The prevalence of HBV infection is high in the Western Pacific and South-East Asia; however, a variable pattern has been observed in different regions of the Middle-East (4). Three areas were proposed on the basis of HBV prevalence in the world (3, 7). The prevalence of hepatitis B virus surface antigen (HBsAg) in hyper-endemic, endemic and hypo-endemic areas is more than 8%, 2% to 7% and less than 2%, respectively (3, 7). The prevalence of HBsAg was reported to be 2-7% in Iran; therefore, Iran was classified as an intermediate HBsAg positive area (7). An estimated 3% of the Iranian population were HBsAg carriers; varying between 1.32% and 6.3% in different regions of the country (8, 9). Vaccination is considered to be the most effective way to control the spread of HBV (10). Recent studies have shown that the changing epidemiology of hepatitis B virus infection in Iran is, at least to some extent, due to HBV vaccination as a national program in routine neonate care. The national expanded HBV vaccination program was implemented in 1993 and 2007 for newborns and adults, respectively (4, 8). In our country, programs such as behavioral interventions, syringe-distribution and vaccination, particularly among male prisoners, are suggested to limit the spread of the virus (11).

## 2. Objectives

The aim of this study was to determine the prevalence of hepatitis B virus among the general population of Mashhad, Iran and also to evaluate anti-HBe and HBe-Ag in HBsAg positive subjects.

## 3. Materials and Methods

Based on multistage cluster-sampling method, 3198 people were recruited from March 2010 to November 2011 for the study of HBV infection in Mashhad. Several procedures were employed to ensure that the studied population represents the entire population of Mashhad. In every district, 9 subdivisions were chosen randomly using proportional population size (PPS). Next, samples were collected haphazardly from blocks in every subdivision. Following the registration of 3198 individuals in the study, informed consents, which were approved by the Ethic’s committee of MUMS, were obtained, and a questionnaire was filled for each individual. Variables included; HBV infection, age, gender, acute HBV infection, chronic HBV infection and the function of HBV infection. A ten ml blood sample was taken from each subject. In the next stage, serum was separated and HBsAg prevalence was determined using ELISA (Delaware Biotech, USA). HBsAg positive sera were subjected for viral DNA extraction using commercial DNA extraction kit (GeNet Bio, Korea) and HBV PCR using the virus specific primers. For PCR amplification, the mixture consisted of 2.5 μl of 10xPCR buffer, 20 pmol of forward (5'-CGGTAAAAAGGGACTCAAGAT-3') and reverse (5'-GTGGTGGACTTCTCTCAATTTTC-3') primers, 1.5 mM of MgCl2, 0.2 mM of each dNTP, 3U of Taq polymerase (CinnaGen, Iran) in a total reaction volume of 25 μl. Amplification was carried out for 35 cycles (95°C for 30 sec, 57°C for 30 sec, 72°C for 1 min) after an initial denaturation step in 95°C for 5 min. The cycles were followed by a 5 min extension at 72°C and the PCR product was visualized on a 2% agarose gel by Green-Viewer staining. Anti-HBc IgM (Delaware Biotech, USA) was utilized to investigate acute and chronic HBV infection in HBsAg positive individuals. Anti-HBe and HBe-Ag ELISA tests (Delaware Biotech, USA) were carried out to assess the replication of HBV in infected individuals. Statistical analysis was performed with Pearson's Chi-square and the data was analyzed by SPSS v.18 (SPSS Inc., Chicago, IL, USA).

## 4. Results

**Figure 1.**
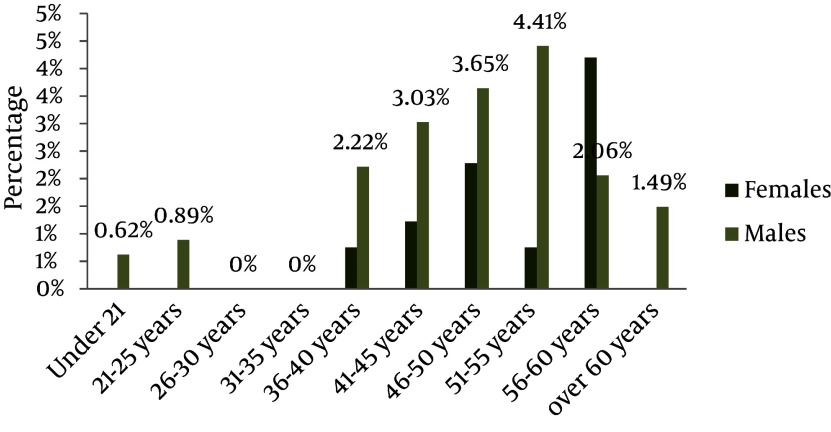
TheFrequency of HBsAg Positivity among Men and Women According to their Age Groups

**Figure 2.**
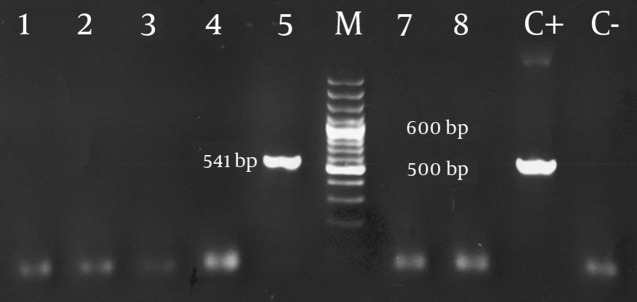
Gel Electrophoresis of PCR Products PCR was performed for amplifying 541 bp fragment using specific primers for the HBV genomic DNA sequences. Lanes 1 to 4 and 7 to 8 correspond to HBV negative PCR and lane number 5 is a HBV PCR positive sample; M = 100 bp DNA size marker; C+ = positive control; C-= negative control of the PCR

Of the 3198 individuals, 2177 (68%) were female and 1021 (32%) were male. The mean age was 38 years old. The prevalence of HBsAg was 0.7% and 1.6% among females and males, respectively. The data displayed a statistically significant correlation between gender and HBsAg frequency, with the prevalence being higher among males (P = 0.029). The data demonstrated an upward trend in prevalence with increasing age and reached its peak of 3% in the 55-60 years age group. In the age group 25-35, the prevalence was zero. The trend indicated a strong association between age and HBsAg infection. Older females showed an elevated risk of HBsAg frequency. The highest prevalence was 2.4% in 50-55 years age group. Among females younger than 35, its prevalence was zero. The highest prevalence among males was 4.5% for the age group 55-60, which is close to that of their total population. HBsAg was zero among males in the 25-35 years age group (Figure 1). HBeAb prevalence in HBsAg-positive individuals was 25%. The same results were observed for males and females when considered separately. HBeAb had its lowest frequency of 20 percent among individuals older than 60. Regarding each gender, the prevalence of HBeAg among HBsAg-positive females was 6.3%. This frequency was zero for HBsAg-positive males. Considering both genders, the prevalence in the total population was 31%. Among HBsAg positive patients, HBc IgM frequency was 3.1%. This frequency was 6.3% and zero among females and males, respectively. The total prevalence of PCR-positive patients was 3.1%, which is similar to previous results. The frequency was 6.3% and zero among females and males, respectively. Results of some HBV PCR tests with specific viral primers are given in Figure 2. Positive samples indicated 541bp fragments.

## 5. Discussion

The prevalence of HBsAg in Middle Eastern countries ranges between 2% and 20% (12). Our study demonstrated that the prevalence of HBsAg in Mashhad was 1.1%. As a result, by definition, this city falls into the hypo-endemic category. A study conducted on blood donors suggested that approximately 35% of Iranians have been in contact with HBV and 3% of them become chronic carriers (13), yet, this study was not a suitable indicator of the total population. HBsAg rate has been reported for different provinces of Iran. Merat et al. showed that in three provinces of Iran, Tehran, Hormozgan and Golestan, the frequency of HBsAg was 2.3%, 2.7% and 5.1%, respectively (9). HBV prevalence in Iran was reported to be 1-2% in 1977 while a rise to 2.49-3.5% was observed between 1988 and 1993. Recent studies have estimated it to be between 1.2 and 9.7% in different provinces (8). According to our findings, consistent with some previous data (14-16), the proportion of men positive for HBsAg was higher, while other studies did not suggest any strong correlation between gender and HBsAg prevalence (17). Our data demonstrated an elevated risk of HBsAg infection among older subjects. This finding has been confirmed in previous studies (16). The prevalence of HBeAg among HBsAg individuals seemed to be lower in our study in comparison with similar studies (16, 18-22). A possible explanation could be mutant forms which were not evaluated in our study. We noted that the lowest prevalence of HBeAb among HBsAg-positive subjects was in the age group 35-40. Our data, contradicts that found by Dow et al. (19) and McMahon (16), which did not support the increase in HBeAb prevalence among older subjects. HBc IgM indicates acute hepatitis B infection. Three percent of HBsAg-positive patients were also positive for anti-HBc IgM. In a study by Kumar et al. 47 out of 2552 blood donors were HBsAg positive, of whom, only one was positive for anti-HBc IgM and the total frequency was 0.43% (23). One significant limitation of our study was not having enough data on medical history of patients such as previous infection with blood-borne diseases like HTLV and HIV, which are prevalent in our region. Our data showed the prevalence of HBV is low in our region. It seems that the national expanded vaccination program was successful, at least among the younger generation, to control the spread of Hepatitis B virus. Recent studies have shown that the prevalence of HBV is remarkably varied in different provinces of Iran. Therefore, follow-up studies are crucial in order to achieve more satisfactory findings and better organized health programs. Regarding the fact that HBV is preventable, such studies may provide a better understanding of the epidemiological pattern of HBV as well as an insight in to the success of vaccination programs in previous years.
